# The use of minimal fluoroscopy for cardiac electrophysiology procedures: A meta‐analysis and review of the literature

**DOI:** 10.1002/clc.23609

**Published:** 2021-05-17

**Authors:** Lorraine Lok Wing Chiang, Christien Li, Kathryn L Hong, Winsy Sin Hui, Sze Yi Beh, Mengqi Gong, Tong Liu, Guangping Li, Yunlong Xia, Jeffery Ho, Leonardo Roever, Sophia Duong, Grace Huang, Gary Tse, Adrian Baranchuk, Benedict M. Glover, International Health Informatics Study (IHIS) Network

**Affiliations:** ^1^ Li Ka Shing Faculty of Medicine University of Hong Kong Hong Kong China; ^2^ Department of Medicine and Therapeutics, Faculty of Medicine Chinese University of Hong Kong Hong Kong China; ^3^ Li Ka Shing Institute of Health Sciences, Faculty of Medicine Chinese University of Hong Kong Hong Kong China; ^4^ Faculty of Medicine Newcastle University Newcastle UK; ^5^ Division of Cardiology University of Toronto Toronto Ontario Canada; ^6^ Tianjin Key Laboratory of Ionic‐Molecular Function of Cardiovascular disease, Department of Cardiology, Tianjin Institute of Cardiology Second Hospital of Tianjin Medical University Tianjin China; ^7^ Department of Cardiology First Affiliated Hospital of Dalian Medical University Dalian China; ^8^ Department of Anaesthesia and Intensive care, Faculty of Medicine Chinese University of Hong Kong Hong Kong China; ^9^ Federal University of Uberlândia Department of Clinical Research Uberlândia Minas Gerais Brazil; ^10^ Division of Cardiology Kingston General Hospital, Queen's University Kingston Ontario Canada

**Keywords:** catheter ablation, fluoroscopy, radiation, X‐ray

## Abstract

**Background:**

Conventional catheter ablation involves prolonged exposure to ionizing radiation, potentially leading to detrimental health effects. Minimal fluoroscopy (MF) represents a safer alternative, which should be explored. Data on the safety and efficacy of this technique are limited.

**Hypothesis:**

Our hypothesis is that MF is of equal efficacy and safety to conventional catheter ablation with the use of fluoroscopy by performing a meta‐analysis of both randomized controlled trials (RCTs) and real‐world registry studies.

**Methods:**

Pubmed and Embase were searched from their inception to July 2020 for RCTs, cohort and observational studies that assessed the outcomes of catheter ablation using a MF technique versus the conventional approach.

**Results:**

Fifteen studies involving 3795 patients were included in this meta‐analysis. There was a significant reduction in fluoroscopy and procedural time with no difference in acute success (odds ratio [OR]:0.74, 95% CI: 0.50–1.10, p = .14), long‐term success (OR:0.92, 95% CI: 0.65–1.31, p = .38), arrhythmia recurrence (OR:1.24, 95% CI: 0.75–2.06, p = .97) or rate of complications. (OR:0.83, 95% CI: 0.46–1.48, p = .65). Additionally sub‐group analysis for those undergoing catheter ablation for atrial fibrillation (AF) did not demonstrate a difference in success or complication rates (OR:0.86, 95% CI: 0.30–2.42, p = .77). Multivariate meta‐regression did not identify the presence of moderator variables.

**Conclusion:**

This updated meta‐analysis demonstrated an overall reduction in procedural and fluoroscopy time for those undergoing a minimal fluoroscopic approach. There was no significant difference in either acute or chronic success rates or complications between a MF approach and conventional approach for the management of all arrhythmias including those undergoing catheter ablation for AF.

## 1 INTRODUCTION

Conventionally, the anatomical localization of catheters has relied on fluoroscopic imaging during catheter ablation for cardiac arrhythmias. It is well known that ionizing radiation is a proven (Class 1) carcinogen, therefore conventional fluoroscopy‐based techniques carry potential risks to both the operator and the patient. Prolonged radiation exposure has been shown to be associated with an increased prevalence of certain malignancies, genetic defects, cataracts, and dermatitis, especially for high‐risk populations such as children, pregnant women, and people who are immunocompromised.^1^, ^2^, ^3^ The current standard practice is to use as low as reasonably achievable (ALARA) levels of radiation as well as lead protection where possible.^4^, ^5^, ^6^ Although electroanatomic mapping has been used in conjunction with fluoroscopic imaging there has been an increasing interest in the use of minimal fluoroscopy (MF). This has been helped with advancements in intracardiac echocardiography (ICE), 3‐D mapping technology and contact force‐sensing catheters.

Given the significant increase in the clinical volume of EP procedures there have been multiple studies about MF published since the last meta‐analysis in 2016 on this topic.^7^ We conducted an updated meta‐analysis to compare the efficacy and safety parameters of MF with conventional fluoroscopically guided procedures for ablation of cardiac arrhythmias. The main outcomes analyzed include fluoroscopy time, radiation dose, ablation time, procedural duration, acute success, long‐term success, complication rates, and recurrence rates. We also performed a meta‐analysis on MF and conventional fluoroscopy in the catheter ablation of atrial fibrillation (AF), including its acute success, long‐term success, complications rates, and recurrence rates. Finally, we review the current state of adoption of MF technology and areas of future work.

## 2 METHODS

### 2.1 Search strategy, inclusion, and exclusion criteria

This study was conducted according to the Preferred Reporting Items for Systematic Reviews and Meta‐Analyses (PRISM) statement.^8^ Ethics was obtained from University of Hong Kong. PubMed and Embase were searched for studies which compared low or zero fluoroscopy to conventional fluoroscopy in the ablation of cardiac arrhythmia. The following search terms were used for both databases: (radiation or X‐ray or fluoroscopy or fluoroscopic or fluoroscopically) and (catheter ablation). The search period was from January 1974 through to July 2020 without language restrictions. Only fully published studies were used. The following inclusion criteria were used: (i) Studies involving patients with cardiac arrhythmia requiring catheter ablation, (ii) difference in outcome between the two procedures, conventional ablation and zero or nonzero fluoroscopy, were compared. These outcomes included fluoroscopic time, radiation dose, ablation time, procedure duration, acute success, long‐term success, complications, or arrhythmia recurrence.

The Newcastle–Ottawa Quality Assessment Scale (NOS) was used for quality assessment of the included studies.^9^ The NOS system evaluated the categories of study participant selection, results comparability, and quality of the outcomes. Specifically, the following characteristics were assessed: (a) Representativeness of the exposed cohort; (b) selection of the nonexposed cohort; (c) ascertainment of exposure; (d) demonstration that outcome of interest was not present at the start of study; (e) comparability of cohorts based on study design or analysis; (f) assessment of outcomes; (g) follow‐up periods that were sufficiently long for outcomes to occur; and (h) adequacy of follow‐up of cohorts. This scale varied from 0 to 9 stars, which indicated that studies were graded as poor quality if the score was <5, fair if the score was 5 to 7, and good if the score was >8. Studies with a score equal to or higher than six were included. The details of the NOS quality assessment are shown in Supplementary Tables 1.

### 2.2 Data extraction and statistical analysis

Data from different studies were entered in pre‐specified spreadsheets in Microsoft Excel. All potentially relevant studies were retrieved as complete manuscripts, which were assessed fully to determine their compliance with the inclusion criteria. The following data were extracted from the included studies: (i) Publication details: last name of first author, publication year, and locations; (ii) study design; (iii) outcomes(s); and (vi) characteristics of the population including sample size, gender, age, and number of subjects. Two reviewers (L. C. and C. L.) reviewed each included study independently. Disagreements were resolved by adjudication with input from a third reviewer (G. T.). Heterogeneity across studies was determined using Cochran's *Q*‐value and the *I* ^2^ statistic from the standard chi‐square test. Cochran's *Q*‐value is the weighted sum of squared differences between individual study effects and the pooled effect across studies. The *I* ^2^ statistic from the standard chi‐square test describes the percentage of variability in the effect estimates resulting from heterogeneity. *I* ^2^>50% was considered to reflect significant statistical heterogeneity. The random‐effects model using the inverse variance heterogeneity method was used with *I* ^2^>50%. To locate the origin of the heterogeneity, sensitivity analysis excluding one study at a time was also performed. Funnel plots showing standard errors or precision against the logarithms of the odds ratio were constructed. The Begg and Mazumdar rank correlation test and Egger's test were used to assess for potential publication bias (Figure 3). Associations between population co‐variables and study outcomes were explored using multivariate meta‐regression. To account for missing data, we used mean imputation (<10% missing) or random imputation (>10% missing). All statistical analysis was conducted using the Review Manager 5.3 for MacOS and Comprehensive Meta‐Analysis (CMA) version 3.0 (Biostat, Inc, Englewood, NJ, USA). Statistical significance was set as p‐value of less than .05.

## 3 RESULTS

**FIGURE 1:**
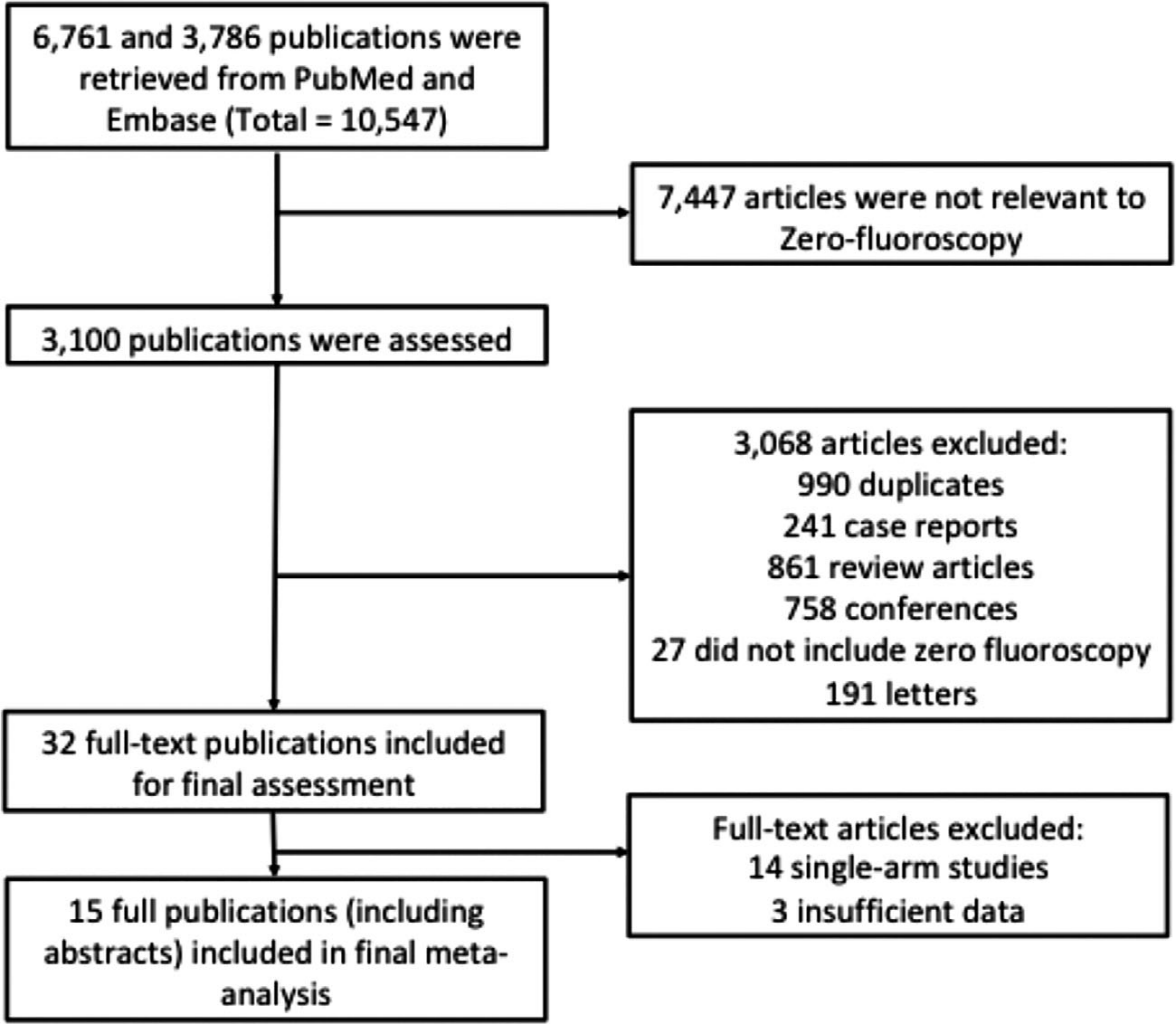
Figure showing the process showing the number of studies retrieved from Pubmed and Embase and how these were selected according to the type of article and inclusion criteria

A total of 15 studies involving 3795 patients met our inclusion criteria and were included in this meta‐analysis. The PRISMA flow chart diagram (Figure 1) shows the study selection process. Of the 15 included publications, 5 were randomized trials while the remaining 10 were non‐randomized studies. All studies included patients with attempted MF ablation. Baseline characteristics of the included studies are summarized in Supplementary Table 2. Overall, this shows that catheter ablations were performed for AVRT, AVNRT, atrial flutter, AF, and VT. The mean follow up ranged from 42 to 389 +/−217 days.

**FIGURE 2:**
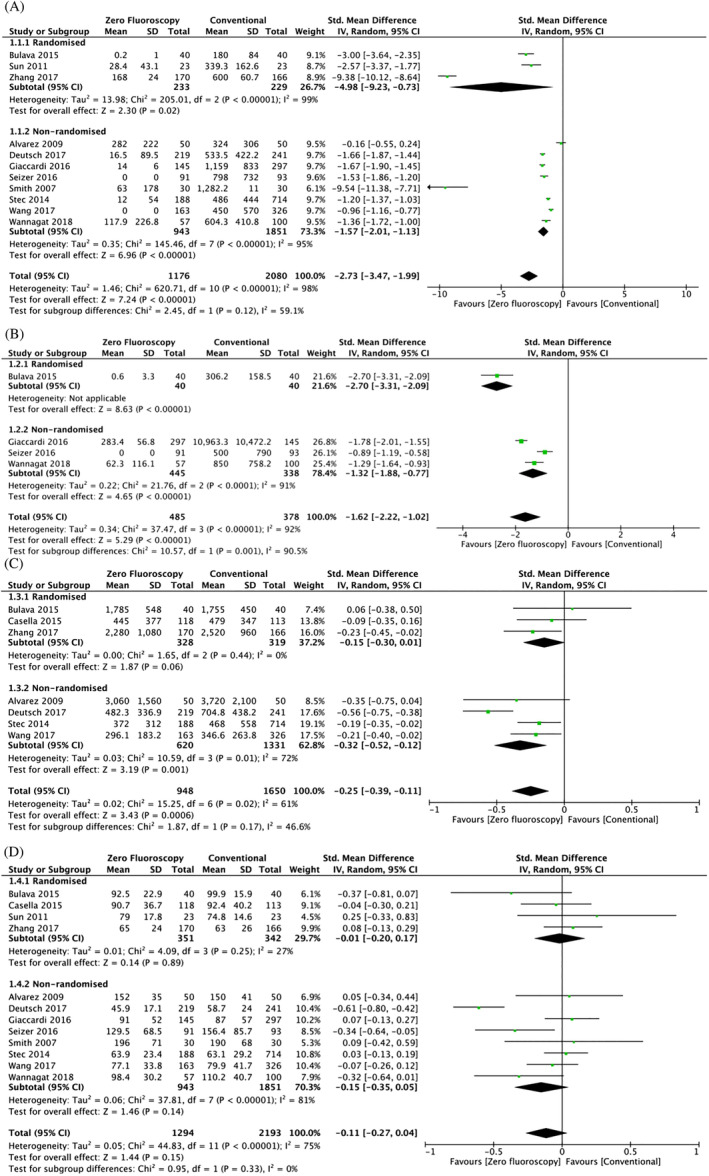
(A) Forest plots demonstrating the changes in fluoroscopic time (s) between zero or near‐zero fluoroscopy with conventional approach. (B) Forest plots demonstrating the changes in radiation dose (cGy/cm^2^) between zero or near‐zero fluoroscopy with conventional approach. (C) Forest plots demonstrating the changes in ablation time (s) between zero or near‐zero fluoroscopy with conventional approach. (D) Forest plots demonstrating the changes in procedural duration (min) between zero or near‐zero fluoroscopy with conventional approach

**FIGURE 3:**
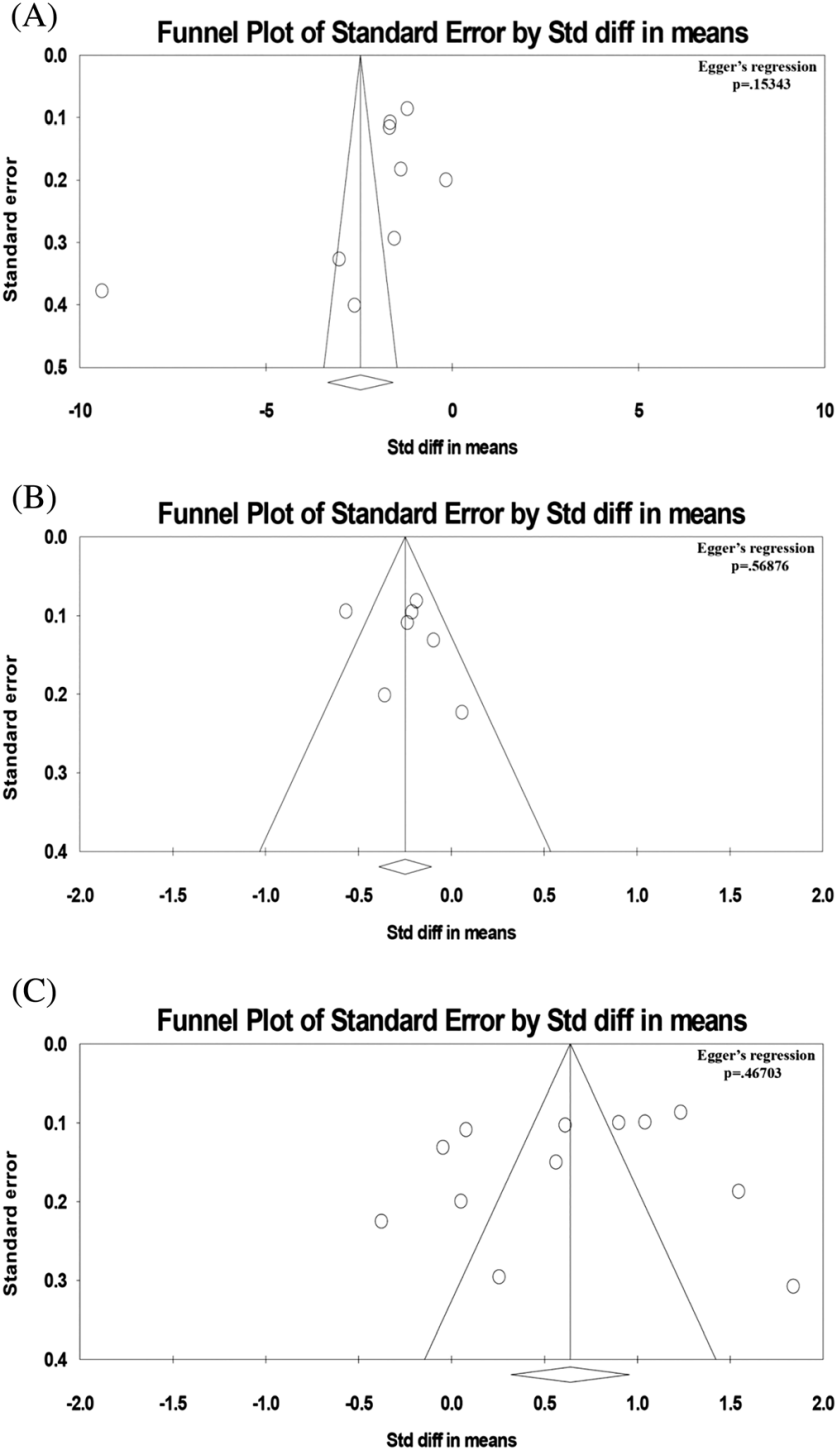
(A) Funnel plot with Egger's regression test in changes in fluoroscopic time (s) between zero or near‐zero fluoroscopy with conventional approach. (B) Funnel plot with Egger's regression test in changes in ablation time (s) between zero or near‐zero fluoroscopy with conventional approach. (C) Funnel plot with Egger's regression test in changes in procedural duration (min) between zero or near‐zero fluoroscopy with conventional approach

The results of our meta‐analysis on fluoroscopic time (available in 11 out of 15 studies), radiation dose (available in 4 out of 25 studies), ablation time (available in 7 out of 25 studies) and procedure duration (available in 9 out of 15 studies) are shown in Figure 2(A)–(D). In these figures, the term 'total' refers to the number of cases recorded Significant reductions in fluoroscopic time, radiation dose and ablation time were observed in the MF group, yielding a standardized mean difference (SMD) of − 2.21 (95% confidence interval [CI]: − 2.89 to − 1.54, p < .001), − 1.62 (95% CI: − 2.22 to − 1.02, p < .001) and − 0.25 (95% CI: − 0.39 to − 0.11, p = .0006), respectively, (Figure 2(A)–(C)). By contrast, mean procedural times were not significantly different between MF and conventional ablation (SMD: − 0.11, 95% CI: − 0.27 to 0.04, p = .15) (Figure 2(D)). Subgroup analyses revealed that fluoroscopic time and radiation dose were significantly reduced for both randomized and non‐randomized studies (Figure 2(A), (B)). However, ablation time and procedural duration were only shorter in non‐randomized studies but not in randomized studies. No significant changes in pooled effects estimates were observed after the trim‐and‐fill adjustment and Egger's tests showed no evidence of publication bias (Egger's regression test p‐values >.05; Figure 3(A)–(C)).

**FIGURE 4:**
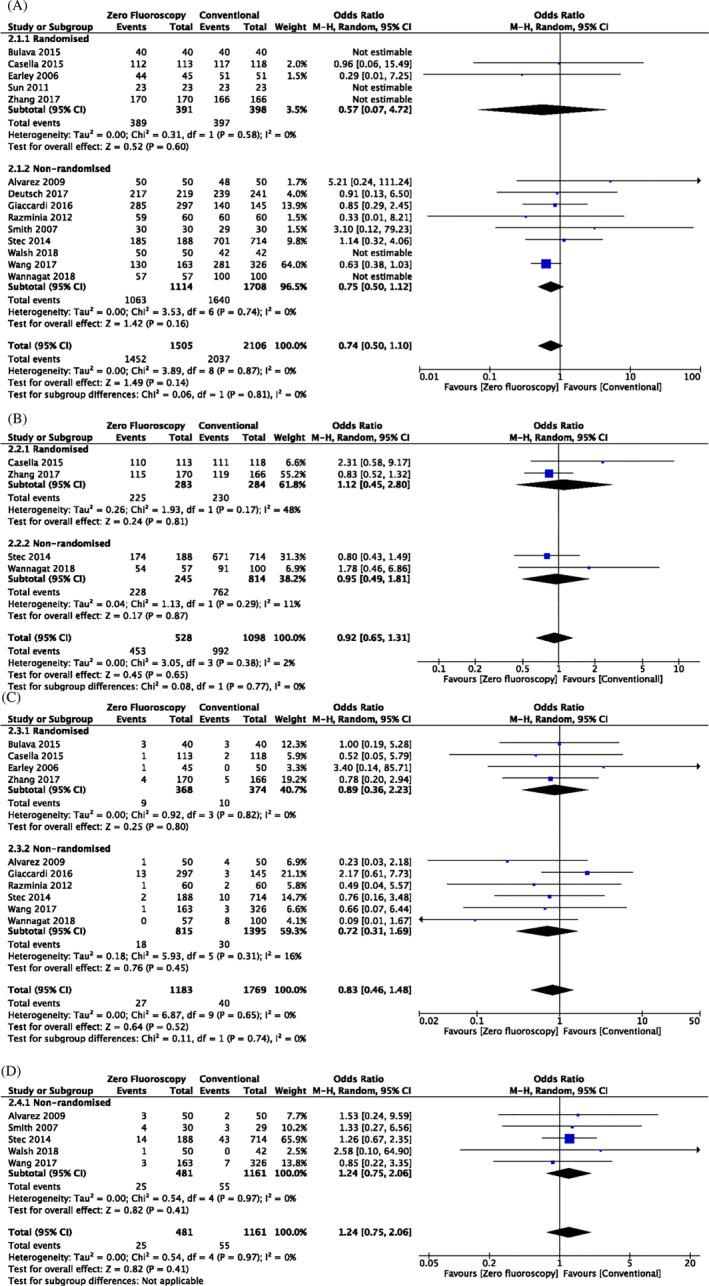
(A) Forest plots comparing the number of acute success between zero or near‐zero fluoroscopy with conventional approach. (B) Forest plots comparing the number of long‐term success between zero or near‐zero fluoroscopy with conventional approach. (C) Forest plots comparing the number of complications between zero or near‐zero fluoroscopy with conventional approach. (D) Forest plots comparing the number of recurrence between zero or near‐zero fluoroscopy with conventional approach

Forest plots for all other clinical outcomes including acute success, long‐term success, complication rate and recurrence rate are shown in Figure 4(A)–(D). The acute success, long‐term success, complication rate, and recurrence rate were recorded in 14, 4, 10, and 5 studies for each outcome, respectively. With the addition of new articles into the meta‐analysis since 2016, there were no significant differences between the MF and conventional groups in terms of acute success (OR: 0.74, 95% CI: 0.50–1.10, p = .14), long‐term success (OR: 0.92, 95% CI: 0.65–1.31, p = .38), complication rates (OR: 0.83, 95% CI: 0.46–1.48, p = .65) or recurrence rates (OR: 1.24, 95% CI: 0.75–2.06, p = .97) (Figure 4(A)–(D)).

**FIGURE 5:**
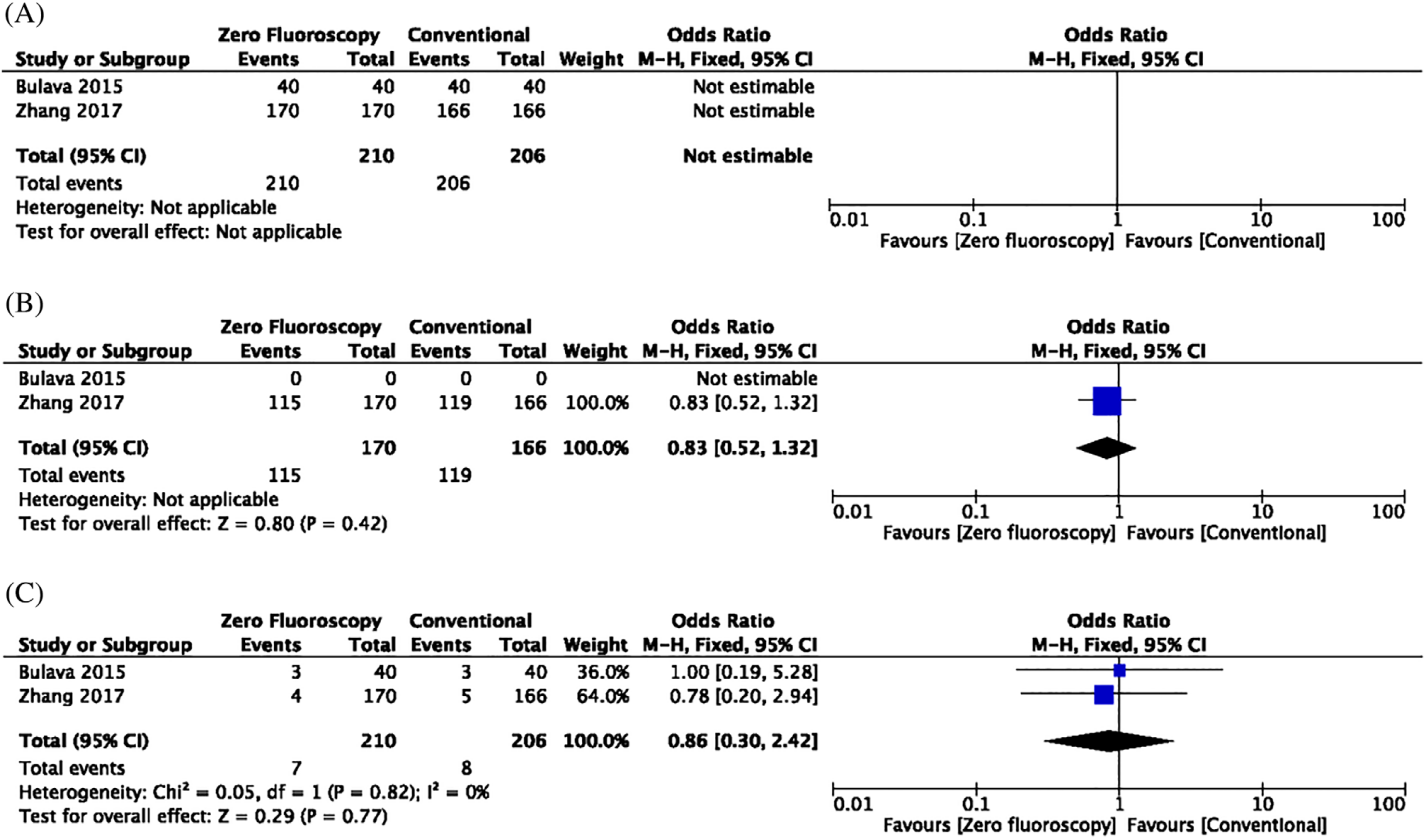
(A) Forest plots comparing the number of acute success between zero or near‐zero fluoroscopy with conventional approach in atrial fibrillation patients. (B) Forest plots comparing the number of long‐term success between zero or near‐zero fluoroscopy with conventional approach in atrial fibrillation patients. (C) Forest plots comparing the number of complications between zero or near‐zero fluoroscopy with conventional approach in atrial fibrillation patients

Of the publications included in this review, two randomized studies exclusively examined AF procedures. A meta‐analysis of these two studies included 416 patients with AF treated either with MF or conventional fluoroscopic techniques. Both studies showed that MF and conventional techniques achieved 100% acute success. Long‐term success rates were only reported and therefore only estimable in Zhang et al. (OR: 0.83, 95% CI: 0.52–1.32, p = .42), which showed no significant difference between the two groups after 1 year. Furthermore, no significant increase in complication rates was observed in the MF experimental group compared to conventional fluoroscopic techniques (OR: 0.86, 95% CI: 0.30–2.42, p = .77) (Figure 5(A)–(D)).

### 3.1 Multivariate regression analysis for outcomes

We conducted a multivariate meta‐regression, using all covariates that were common across any of the 15 studies that included the three outcomes: acute success, complication rates, and recurrence rates. Long‐term success as an outcome was omitted from the multivariate regression due to lack of reporting from the included articles. Covariates used were mean population age, male gender, fluoroscopy time, ablation time, and procedure time. Both acute success and complication outcomes utilized all covariates while the recurrence rate outcome included only two covariates (fluoroscopy time and ablation time) for multivariate analysis (Supplementary Table 3). None of the covariates tested significantly moderated the acute success, complication rate and recurrence rate outcomes. Beta‐coefficients for each covariate did not differ significantly from zero other than male gender (p > .05).

## 4 DISCUSSION

There has been an increased interest in performing MF techniques particularly since the last meta‐analysis was published on this topic.^7^ This study provides an update to a previously published meta‐analysis by including five additional studies (one randomized and four non‐randomized studies).^10^, ^11^, ^12^, ^13^, ^14^ In total, we included 15 clinical studies involving 3795 patients who underwent catheter ablation with MF or conventional fluoroscopy for cardiac arrhythmias. Our results demonstrate a significant reduction in fluoroscopic time, radiation dose and ablation time in the MF group with no difference in total procedural time, short‐term success, long‐term success, complication rates, and recurrence rates. This is a very important finding as the use of MF is not associated with an increase in the duration of the procedure, success rates and most important rate of complications. This should be encouraging for operators who are looking at adopting this approach.

Furthermore, Zhang et al. and Bulava et al. were meta‐analyzed to compare the two techniques specifically for AF (AF) procedures.^12^, ^15^ The two outcomes analyzed—acute success and complication rates, did not differ significantly between the two approaches.

Subsequently, multivariate meta‐regression was conducted on common covariates, including mean age, male gender, fluoroscopy time, ablation time, and procedural time for all outcomes except for long‐term success. This analysis showed that these variables had no impact on acute success, complication and recurrence outcomes.

### 4.1 Cancer risk associated with fluoroscopy and operator preference

There is sparse evidence regarding the long‐term radiation effects in interventional‐cardiac electrophysiology, thus emphasizing the need for additional studies in this area.^16^, ^17^ In a study by Casella et al., they estimated that a switch from a fluoroscopic to MF approach would result in a 96% reduction in the estimated risks of cancer incidence, mortality, estimated years of life lost and years of life affected.^18^

The use of MF approaches becomes especially important in children, pregnant women and women of childbearing potential.^19^, ^20^ In a survey published in 2017 by the European Society of Cardiology, the percentage of female trainees in electrophysiology is only 11%, with female cardiologists making more changes in their training and careers to reduce or avoid radiation exposure because of concerns related to risk to a developing fetus.^21^

Furthermore, it also appears that there is a higher incidence of sperm DNA fragmentation and hyper‐methylated spermatozoa, suggesting that occupational exposure has substantial detrimental effects on sperm function, genetic and epigenetic integrity in healthcare workers.^22^ Unsurprisingly, surveys have shown that healthcare professionals favored the zero fluoroscopy approach over the conventional approach.^10^, ^23^ Even though the use of lead aprons is useful in reducing radiation exposure, it has been shown to only effectively reduce one‐third of the scattered radiation^27^ Wang et al. also observed lower mean fatigue score with the zero fluoroscopy approach as compared to the conventional group (2.1 ± 0.7 vs. 3.9 ± 1.6, p < .05),^10^ which may partially be due to the use of lead aprons. This data underscores the importance of both development and adoption of MF technology for the field of electrophysiology in terms of reducing occupational health burdens on healthcare workers.

### 4.2 Zero fluoroscopy in specific arrhythmias

Finally, it is worth noting that 13 out of 15 studies included in this meta‐analysis included a broad range of arrhythmias ranging from atrial flutter to idiopathic ventricular arrhythmias, as illustrated in Supplementary Table 2. Considering two studies with similar patient populations,^24^, ^25^ Alvarez et al. only used AVNRT patients while Razminia et al. utilized a population with a wide arrhythmic background. This resulted in a significantly different odds ratio in acute success favoring different ablation approaches. Furthermore, Walsh et al. reported significantly less ablation time was required to achieve cavo‐tricuspid isthmus block in typical atrial flutter using MF. This may reflect the fact that three dimensional electroanatomical mapping may increase the accuracy of the ablation lesions compared with fluoroscopy making it more straightforward for the operator to ascertain any potential conduction gaps. Minimizing ablation time and instances might have the potential to reduce ablation‐related complications.^14^ As such, the utility of MF over conventional ablation cannot be confirmed in arrhythmia‐specific instances.

### 4.3 Current barriers to adoption–cost and training

Currently available data suggest that the use of MF technique for individual patients is predicted by the type of arrhythmia, operator experience and patient's age.^26^ Another common concern is the difficulty of learning to use a different modality. However, studies have shown that the MF learning curve is not unreasonably steep with the learning burden occurring over the first 20, 15, and 40 cases for SVT, PVC, and PVI ablation, respectively.^27^ Furthermore, in a study by Gist et al. they found that procedure time significantly shortens as a function of experience, reaching acceptable time frames in comparison to current fluoroscopic approaches after 12 months of use.^28^ Despite such benefits, higher cost incurred with zero fluoroscopy has been previously reported.^29^ This issue was elegantly addressed by Casella et al., whose analysis found that the additional cost of MF technology is approximately equal to the extra costs associated with increased cancer treatment and reduction in quality of life associated with traditional fluoroscopic techniques.^18^

### 4.4 Current and future directions

Currently, through this meta‐analysis, we found no significant difference in outcome measures between conventional and MF techniques. This is a reassuring finding, confirming a previous important study.^7^ It would be beneficial for future studies to compare the two approaches in specific arrhythmia types. Moving forward, subgroup analysis of the effectiveness of MF approaches to different procedures, which vary widely in their baseline radiation exposure and technical difficulty, would be useful to inform practitioners that specialize in specific procedures. Long‐term follow‐up studies should focus on long‐term patient outcomes and healthcare use experiences to better elucidate how to implement MF technology into established practices.

## 5 LIMITATIONS

Several limitations of this study should be noted. First, a high degree of heterogeneity is still observed (>50%) between the different study populations. This was addressed by conducting a sensitivity analysis excluding one study at a time along with Egger's test, which overall showed a nonsignificant publication bias. A multivariate meta‐regression analysis also confirmed that patient characteristics and procedural parameters have no impact on outcome measures. Second, abstracts presented at scientific meetings and non‐English studies were excluded from this meta‐analysis, which may result in a small degree of selection bias. Finally, all studies listed the arrhythmia subtypes that were included in the comparison (Supplementary Table 2) but only two authors (Earley, et al. and Smith, et al.) went on to briefly specify the complications with each subtype. As such, a sub‐analysis of each arrhythmia subtype was not possible.

## 6 CONCLUSIONS

This systematic review and meta‐analysis confirmed that the use of MF does not result in an increase in procedural time, success rates or complication rates when compared with conventional techniques. These parameters were also not significantly different for patients undergoing catheter ablation for AF. This is highly encouraging data for those who are seeking to adopt this technique for the management of cardiac arrhythmias.

## CONFLICT OF INTEREST

Authors declare no potential conflict of interest.

## Supporting information


**Supplementary Table 1** NOS risk of bias scale for included cohort studies

**Supplementary Table 2** Characteristics of the 19 studies included in this meta‐analysis

Supplementary Table 3

## Data Availability

The data that support the findings of this study are available from the corresponding author upon reasonable request.
