# Supplementary material for: The use of minimal fluoroscopy for cardiac electrophysiology procedures: A meta‐analysis and review of the literature
Source: Clin Cardiol. 2021 May 17;44(6):814–23. doi: 10.1002/clc.23609 (PMC8207968; doi:10.1002/clc.23609)
Supplement: Supplementary file 1 — Supplementary Table 1 NOS risk of bias scale for included cohort studies [file CLC-44-814-s002.docx]

NOS risk of bias scale for included cohort studies

|  |  | Selection |  |  |  |  | Outcome |  |  |
| --- | --- | --- | --- | --- | --- | --- | --- | --- | --- |
| Studies | Representativeness of the exposed cohort | Selection of the non-exposed cohort | Ascertainment of exposure | Outcome of interest not present at start of study | Comparability | Assessment of outcome | Adequacy of duration of follow-up | Adequacy of completeness of follow-up | Total score  (0-9) |
| Alvarez 2009 | 1 | 1 | 1 | 1 | 1 (age) | 1 | 1 | 1 | 8 |
| Deutsch 2017 | 1 | 1 | 1 | 1 | 1 (age) | 1 | 1 | 1 | 8 |
| Giaccardi 2016 | 1 | 1 | 1 | 0 | 1 (age) | 1 | 1 | 1 | 7 |
| Razminia 2012 | 1 | 1 | 1 | 0 | 1 (age) | 1 | 1 | 1 | 7 |
| Seizer 2016 | 1 | 1 | 1 | 0 | 1 (age) | 1 | 1 | 1 | 7 |
| Smith 2007 | 1 | 1 | 1 | 0 | 1 (age) | 1 | 1 | 1 | 7 |
| Stec 2014 | 1 | 1 | 1 | 1 | 0 | 1 | 1 | 1 | 7 |
| Walsh 2018 | 1 | 1 | 1 | 0 | 1 (age) | 1 | 1 | 1 | 7 |
| Wang 2017 | 1 | 1 | 1 | 1 | 1 (age) | 1 | 1 | 1 | 8 |
| Wannagat 2018 | 1 | 1 | 1 | 0 | 2 (age, LVEF) | 1 | 1 | 1 | 8 |
