# Supplementary material for: The use of minimal fluoroscopy for cardiac electrophysiology procedures: A meta‐analysis and review of the literature
Source: Clin Cardiol. 2021 May 17;44(6):814–23. doi: 10.1002/clc.23609 (PMC8207968; doi:10.1002/clc.23609)
Supplement: Supplementary file 2 — Supplementary Table 2 Characteristics of the 19 studies included in this meta‐analysis [file CLC-44-814-s003.docx]

**Table 1** Characteristics of the nineteen studies included in this meta-analysis

| First author / Year | Study design | Sample size (n) | Number in experimental group | Number in control group | Arrhythmia included | Outcomes | Follow-up duration (days) |
| --- | --- | --- | --- | --- | --- | --- | --- |
| Bulava 2015 | R | 80 | 40 | 40 | AF | FT, RD, AbT, AS, C | 365 |
| Casella 2015  (no) | R | 231 | 118 | 113 | AVNRT, Right AP, Left AP, AFL, AT | AbT, AS, LTS, C | 365 |
| Earley 2006  (yes) | R | 96 | 45 | 51 | AVNRT, AVRT, AFL, Other | AS, C | 42 |
| Sun 2011  (no - missing) | R | 46 | 23 | 23 | AVNRT, AVRT, AFL, Other | FT, AS | 182.5 |
| Zhang 2017 | R | 336 | 170 | 166 | Paroxysmal AF | FT, AbT, PD, AS, LTS, C | 365 |
| Alvarez 2009 | NR | 100 | 50 | 50 | AVNRT | FT, AbT, PD, AS, C, Re | 182.5 |
| Deutsch 2017 | NR | 460 | 219 | 241 | CTI-dependent AFL | FT, AbT, PD, AS, C | - |
| Giaccardi 2016  (no – missing) | NR | 442 | 297 | 145 | AVNRT, Right AFL, AP, AT, VT, other | FT, RD, PD, AS | - |
| Razminia 2012  (not used) | NR | 120 | 60 | 60 | AF, AFL, AT, AVNRT, AVRT, VT | AS, C | 150 |
| Seizer 2016  (no – missing) | NR | 184 | 91 | 93 | AVNRT, WPW, AT, Typical flutter | FT, RD, PD | 389 ± 217 |
| Smith 2007  (yes – missing) | NR | 60 | 30 | 30 | AVNRT, WPW, concealed pathway | FT, PD, AS, Re | 90, 365 |
| Stec 2014  (yes incomplete – missing) | NR | 902 | 188 | 714 | AVNRT, WPW/OAVRT, AFL, AT | FT, AbT, PD, AS, LTS, C, Re | 330 ± 171 |
| Walsh 2018  (no) | NR | 92 | 50 | 42 | AVNRT, AFL, AP, AT | AS, Re | 149 (Median) |
| Wang 2017  (n0) | NR | 489 | 163 | 326 | VT, Premature ventricular contractions | FT, AbT, PD, AS, C, Re | 164.25 |
| Wannagat 2018  (no) | NR | 157 | 57 | 100 | AF, AFL, VT ]  (btw, this study included AF, should we include it in the AF analysis???) | FT, RD, PD, AS, LTS, C | 91.25 |

Abbreviations: R: Randomised; NR: Non-randomised; AF: Atrial fibrillation; AVNRT=……….; AVRT=…….; AFL: Atrial flutter; AP:…………; AT: Atrial tachycardia; WPW:…………….; OAVRT: …………..; CTI =……………..; VT: Ventricular tachycardia; FT: Fluoroscopic time; RD: Radiation dose; AbT: Ablation time; PD: Procedure duration; AS: Acute success; LTS: Long-term success; C: Complications; Re: Recurrence
