# Supplementary material for: The use of minimal fluoroscopy for cardiac electrophysiology procedures: A meta‐analysis and review of the literature
Source: Clin Cardiol. 2021 May 17;44(6):814–23. doi: 10.1002/clc.23609 (PMC8207968; doi:10.1002/clc.23609)
Supplement: Supplementary file 3 — Supplementary Table 3 [file CLC-44-814-s001.docx]

| Multivariate Meta-Regression | | | | | | |
| --- | --- | --- | --- | --- | --- | --- |
| Variable | Coefficient ($\beta$) | SE | Z-value | P-value | 95% CI | |
|  |  |  |  |  | Lower Limit | Upper Limit |
| Acute Success | | | | | | |
| Mean Age (years) | -0.065 | 0.117 | -0.55 | 0.579 | -0.293 | 0.164 |
| Male Gender | 1.274 | 4.686 | 0.27 | 0.786 | -7.911 | 10.458 |
| Fluoroscopy time (min) | 0.070 | 0.148 | 0.47 | 0.638 | -0.221 | 0.360 |
| Ablation time (sec) | 0.001 | 0.002 | 0.83 | 0.405 | -0.002 | 0.004 |
| Procedure duration (min) | -0.020 | 0.047 | -0.43 | 0.670 | -0.112 | 0.072 |
| Complications | | | | | | |
| Mean Age (years) | -0.022 | 0.081 | -0.27 | 0.783 | -0.081 | 0.137 |
| Male Gender | 3.220 | 5.399 | 0.60 | 0.551 | -7.362 | 13.801 |
| Fluoroscopy time (min) | 0.141 | 0.114 | 1.24 | 0.216 | -0.083 | 0.364 |
| Ablation time (sec) | 0.0001 | 0.001 | 0.23 | 0.817 | -0.001 | 0.001 |
| Procedure duration (min) | -0.005 | 0.022 | -0.23 | 0.817 | -0.049 | 0.039 |
| Recurrence | | | | | | |
| Fluoroscopy time (min) | 0.041 | 0.159 | 0.26 | 0.798 | -0.272 | 0.353 |
| Ablation time (sec) | 0.0001 | 0.0003 | 0.34 | 0.732 | -0.001 | 0.001 |
